# Aerosol emission in professional singing of classical music

**DOI:** 10.1038/s41598-021-93281-x

**Published:** 2021-07-21

**Authors:** Dirk Mürbe, Martin Kriegel, Julia Lange, Hansjörg Rotheudt, Mario Fleischer

**Affiliations:** 1 Department of Audiology and Phoniatrics, Charité-Universitätsmedizin Berlin, corporate member of Freie Universität Berlin and Humboldt-Universität zu Berlin, Berlin, 10117 Germany; 2 Technische Universität Berlin, Hermann-Rietschel-Institut, Berlin, 10587 Germany

**Keywords:** Medical research, Epidemiology

## Abstract

In this study, emission rates of aerosols emitted by professional singers were measured with a laser particle counter under cleanroom conditions. The emission rates during singing varied between 753 and 6093 particles/sec with a median of 1537 particles/sec. Emission rates for singing were compared with data for breathing and speaking. Significantly higher emission rates were found for singing. The emission enhancements between singing and speaking were between 4.0 and 99.5 with a median of 17.4, largely due to higher sound pressure levels when singing. Further, significant effects of vocal loudness were found, whereas there were no significant differences between the investigated voice classifications. The present study supports the efforts to improve the risk management in cases of possible aerogenic virus transmission, especially for choir singing.

## Introduction

The respiratory system is the main transmission route for airborne viruses, which is of particular interest at present as SARS-CoV-2 viruses cause life-threatening COVID-19 disease^1^. It has been shown that patients with COVID-19 disease produce aerosol particles containing infectious viruses, detectable up to 4.8 m away from the patient^2^. Virus-laden aerosol particles are detected in hospital rooms, despite mechanical ventilation with 12 air changes per h^3^. Aerosol particles are responsible for the rapid transmission of pathogen viruses in enclosed rooms^4^ which necessarily involves airborne isolation precautions for safety purposes^5^.

When aerosols are exhaled, the fluid component of the pathogen-containing particles evaporates. The particles become lighter, can float in the air for longer periods^6^ and spread in enclosed rooms by airflow and turbulent diffusion^7^. The efficient removal of viruses within these enclosed rooms is critically determined by the ventilation design^8^. As the basis of an aerogenic transmission of the SARS-CoV-2-virus, the spatial distribution of aerosols is dependent on several factors of the surrounding air, such as temperature and humidity^9^.

Aerosols and droplets are also produced during speaking and singing, because the respiratory tract has a dual function: the respiratory tract is not only the main tool for ventilation, but also the source of voice and spoken language production. Particle formation in the pulmonary alveoli^10^, flow effects of the vibrating vocal folds, and adjustments of the articulation instruments are regarded as aerosol generating mechanisms^11^.

In comparison to breathing, increased formation of aerosols is found for speaking. Additionally, the number of emitted particles depends on vocal loudness^12^. Recently, using an aerodynamic particle sizer, higher particle emission rates have been found for singing compared to speaking^13^, and especially for singing compared to speaking at higher volumes^14^. As a main result of these two studies, the higher rates are more related to loudness rather than the tasks themselves. Further, high infection rates during indoor choir rehearsals have been reported^15–17^.

Previous measurements focus on fluid mechanical aspects in the near-field plume of the mouth during singing^18,19^. The spread of the emitted droplets is investigated, hence distance rules can be derived for protection against droplet infection. However, a risk assessment including the distribution of aerosols in larger rooms is not possible with this method.

The current investigations aim to initially determine the number and size distribution of small particles emitted in the room by professional singers during singing under cleanroom condition, using a laser particle counter. This information can be the basis for a numerical calculation of the distribution of aerosols in larger rooms, which takes into account the boundary conditions being typical for concert and opera performances. The present data may contribute to improved risk management strategies in the fields of culture and education. They should be used for specification of hygiene measures and ventilation concepts in order to facilitate performances and events.

## Results

### Particle size distribution

**Figure 1:**
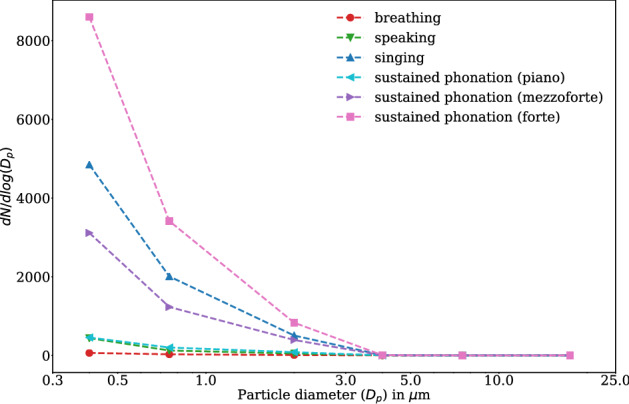
Normalized emission rates for all test conditions according to the legend. Data were averaged across all eight participants and all five replicants for each test condition. Marker positions are at the mean of the particle diameter of each size class on the linear scale (see “Methods” section). Regardless of the test condition, $> 99$% of all detected particles are $\le 5$ µm. The terminology, piano, mezzo-forte, and forte describes singers’ loudness conditions, namely soft, medium, and loud phonation. (Figure created with matplotlib 3.2.1, http://matplotlib.org).

The particle count measurement method detects different sizes of particles from 0.3 to 25 µm. As derived from the particle density distribution plot (Fig. 1), $>99$% of all detected particles were $\le 5$ µm ($> 80$% of all particles $\le 1$ µm). Moreover, the shape of the size distributions is nearly independent of the test conditions.

### Experiment I

**Figure 2:**
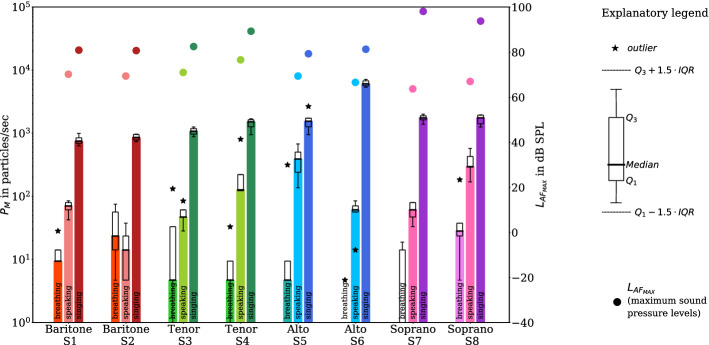
Boxplots of the emission rates (left y-axis; bars represent the median for all replications of each task) for the test conditions breathing, speaking, and singing (denoted by hue and labeled at the bars) and the different voice classifications (baritone (red), tenor (green), alto (blue), soprano (magenta), as denoted in the x-axis) in experiment I. The emission rates for all particle size classes are cumulatively summarized. The black-framed boxes extend from the lower (*Q*1) to upper (*Q*3) quartile values of the data, with a thick line at the median. The lower and upper whiskers represent data greater than $Q1-1.5\cdot (Q3-Q1)$ and lower than $Q3+1.5\cdot (Q3-Q1)$, and stars denote outliers, respectively. For the test conditions speaking and singing, the median of the maximum sound pressure levels $L_{AF_{MAX}}$ for all replications were denoted by full circles (values corresponds to the right y-axis). For breathing, the sound initiated by the technical equipment such as fans was higher than any acoustic sound expelled by the participants. Therefore, no $L_{AF_{MAX}}$ are given. (Figure created with matplotlib 3.2.1, http://matplotlib.org).

Figure 2 illustrates both the emission rates $P_M$ for the different test conditions breathing, speaking, and singing and the median of maximum sound pressure levels for speaking and singing. The results confirm the previous observations of higher emission rates for singing compared to breathing and speaking^14^.

While the median values of all five replications for singing ranged from 753 particles/sec (S1) to 6093 particles/sec (S6) with a median of 1537 particles/sec, those for speaking ranged from 14 particles/sec (S2) to 391 particles/sec (S5) with a median of 66 particles/sec (Table 1). The individual median values for breathing ranged from 0 particles/sec (S6 and S7) to 28 particles/sec (S8) with a median of 5 particles/sec (Table 1).

**Table 1:** Minimum, maximum, and median values of emission rates in particles/sec for breathing, speaking, and singing based on five test replications per test condition (cumulative values for all size classes $>0.3$ µm).

ID	Breathing			Speaking			Singing		
	Min	Median	Max	Min	Median	Max	Min	Median	Max
S1	0	9	28	42	71	85	631	753	998
S2	5	24	75	5	14	38	735	862	970
S3	0	5	132	28	47	85	881	1078	1257
S4	0	5	33	122	127	805	946	1521	1695
S5	5	5	315	137	391	678	951	1554	2665
S6	0	0	5	14	61	85	5368	6093	7171
S7	0	0	19	33	61	80	1398	1761	2011
S8	5	28	184	170	297	574	1257	1761	1959
Median		5			66			1537	

The enhancement of the emission rates for singing in comparison to speaking was between 4.0 (S5) and 99.5 (S6) (median of 17.4). Moreover, the enhancement of the emission rates for singing in comparison to breathing was between 36.6 (S2) and 329.9 (S5) with a median of 154.5 (Table 2).

**Table 2:** Ratios of medians of emission rates for different test and loudness conditions.

ID	Speaking/breathing	Singing/breathing	Singing/speaking	Forte/piano	Forte/mezzo-forte
S1	7.5	80.0	10.7	0.8	1.3
S2	0.6	36.6	61.0	3.4	1.5
S3	10.0	228.9	22.9	121.0	1.6
S4	27.0	322.9	12.0	44.5	6.4
S5	83.0	329.9	4.0	86.0	6.6
S6	Inf	Inf	99.5	159.0	1.5
S7	Inf	Inf	28.8	114.3	2.5
S8	10.5	62.3	5.9	42.0	6.0

The inf-values indicates a non-defined ratio caused by zero values in breathing in the denominator.

Statistical analysis by means of linear mixed modeling showed significant differences of the log-transformed emission rate ${\text {log}}_{10}P_M$ between the different test conditions breathing, speaking, and singing. Condition affected ${\text {log}}_{10}P_M$, increasing it by about $0.78 \pm 0.09$ (standard errors) from breathing to speaking (increases by a ratio of 6.0) and by about $1.21 \pm 0.09$ (standard errors) from speaking to singing (increases by a ratio of 16.2). Both observations were statistically significant (p $<0.001$). Voice classification also affected ${\text {log}}_{10}P_M$, increasing it by about $0.17 \pm 0.19$ (standard errors) from baritone to tenor (increases by a ratio of 1.5), by about $0.12 \pm 0.19$ (standard errors) from tenor to alto (increases by a ratio of 1.3), and decreasing it by about $-0.006 \pm 0.19$ (standard errors) from alto to soprano (with a ratio of about 1.0). These effects were not statistically significant. The intraclass correlation coefficient (ICC) for the cohort was about 15%.

The evaluation of the median of the frequency-weighted (A-weighted) maximum sound pressure level $L_{AF_{MAX}}$ showed higher sound pressure levels for singing (79.3–98.1 db SPL) compared to speaking (63.7–76.6 db SPL). The voice classifications soprano (93.8 and 98.1 db SPL) and tenor (82.5 and 89.3 db SPL) had higher sound pressure levels for singing compared to the altos (79.3 and 81.3 db SPL) and baritones (80.7 and 80.9 db SPL). While the maximum sound pressure level of baritones and tenors in the selected sample were always positively correlated with the particle emission rates, there was no clear correlation in this respect for the altos and sopranos.

### Experiment II

**Figure 3:**
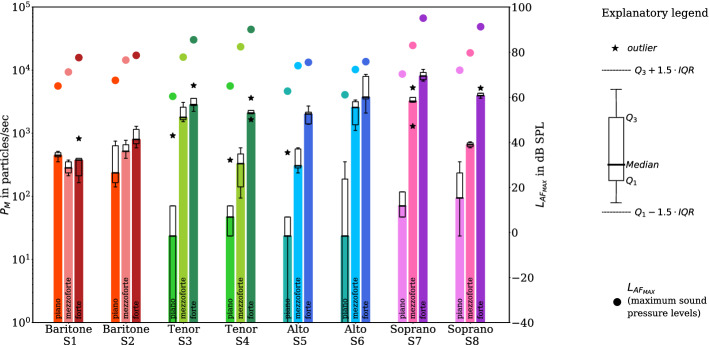
Boxplots of the emission rates (left y-axis; bars represent the median for all replications of each task) while sustaining the vowel /aː/ in experiment II for the different vocal loudness conditions piano, mezzo-forte, and forte and voice classifications (baritone (red), tenor (green), alto (blue), soprano (magenta), as denoted in the x-axis). For the different loudness conditions, the medians of maximum sound pressure levels $L_{AF_{MAX}}$ are also shown (full circles, right y-axis). All symbols and colors correspond to the detailed description given in Fig. 2. (Figure created with matplotlib 3.2.1, http://matplotlib.org) .

The results of the measurements with sustained vowel /aː/ at the different loudness conditions piano, mezzo-forte, and forte are presented in Fig. 3. Seven of the eight participants showed an increase in the emission rate with increasing loudness. This behavior is the weakest for baritones, the lowest vocal range considered in this study. Even more, the emission rates of baritone S1 show no dependence on the test condition.

While the median values of all five replications for piano ranged from 24 particles/sec (S3, S5 and S6) to 447 particles/sec (S1) with a median of 59 particles/sec, those for mezzo-forte ranged from 283 particles/sec (S1) to 3225 particles/sec (S7) with a median of 589 particles/sec. The individual median values for forte ranged from 377 particles/sec (S1) to 8075 particles/sec (S7) with a median of 2472 particles/sec (Table 3).

**Table 3:** Minimum, maximum, and median values of emission rates in particles/sec for piano, mezzo-forte, and forte based on five test replications per test condition (cumulative values for all size classes $>0.3$ µm).

ID	Piano			Mezzo-forte			Forte		
	Min	Median	Max	Min	Median	Max	Min	Median	Max
S1	353	447	518	212	283	377	165	377	824
S2	141	235	753	400	518	777	589	800	1295
S3	0	24	918	1530	1789	3084	2237	2849	5721
S4	24	47	377	94	330	589	1648	2095	3626
S5	0	24	494	235	306	589	1389	2025	2707
S6	0	24	353	1107	2566	3367	2095	3743	8617
S7	47	71	118	1295	3225	5274	6733	8075	10312
S8	24	94	353	589	659	730	3602	3955	5203
Median		59			589			2472	

The comparison of singing piano and forte (Table 3) showed an emission enhancement up to 159.0 (S6) (Table 2). There were higher emission rates for singing in forte for alto and soprano (from 2025 particles/sec (S5) to 8075 particles/sec (S7)) compared to baritone and tenor (from 377 particles/sec (S1) to 2849 particles/sec (S3)). Similarly, in seven out of eight participants, there was an emission enhancement from piano to mezzo-forte (see also Table 2).

Statistical analysis by means of linear mixed modeling showed statistically significant differences of the emission rate ${\text {log}}_{10}P_M$ for the different vocal loudness conditions piano, mezzo-forte, and forte. Vocal loudness affected ${\text {log}}_{10}P_M$, increasing it by about $0.80 \pm 0.09$ (standard errors) from piano to mezzo-forte (increases by a ratio of 6.3) and by about $0.45 \pm 0.09$ (standard errors) from mezzo-forte to forte (increases by a ratio of 2.8). Both observations were significant (p<.001). Voice classification again affected ${\text {log}}_{10}P_M$, increasing it by about $0.09 \pm 0.20$ (standard errors) from baritone to tenor (increases by a ratio of 1.2), by about $0.02 \pm 0.20$ (standard errors) from tenor to alto (with a ratio of about 1.0), and by about $0.24 \pm 0.20$ (standard errors) from alto to soprano (increases by a ratio of 1.7). These effects were not significant. The intraclass correlation coefficient (ICC) for the cohort was also about 15% for experiment II.

**Figure 4:**
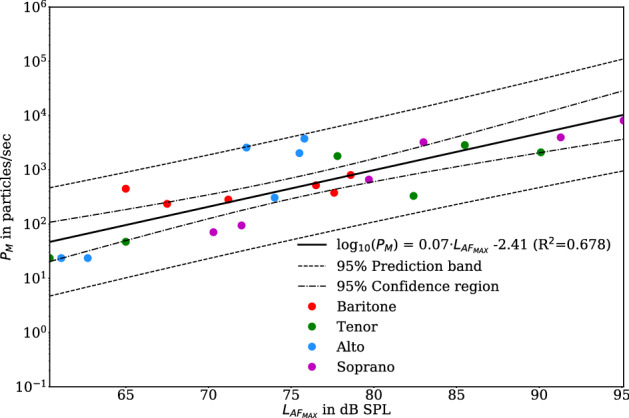
Relationship between emission rate $P_M$ and the maximum sound pressure level $L_{AF_{MAX}}$ for the test conditions of sustained vowel /aː/ (Experiment II) for all three loudness conditions separated by voice classification including linear regression of the logarithmic emission rates (black line). For regression analysis, only medians of $P_M$ and $L_{AF_{MAX}}$ of the five replications were used. (Figure created with matplotlib 3.2.1, http://matplotlib.org) .

For all participants, the intended increase in loudness from piano to forte was reflected in the measured values of the sound pressure level. Additionally, Fig. 4 shows the relationship between the emission rate and the maximum sound pressure level (only the median values of all five replications for experiment II—sustained vowel /aː/—were considered). An increase in the sound pressure level was accompanied by a mean increase in the emission rate ${\text {log}}_{10}P_M$ of about 0.07. More precisely, an increase of 1 dB in $L_{AF_{MAX}}$ leads to an increase by a factor of 1.17 particles/sec in the linear scaled $P_M$. Further, concerning sustained vowels, the emission rates can vary by more than two orders of magnitude between participants and condition.

## Discussion

Due to the increased risk of transmission of SARS-CoV-2 viruses during singing and the described accumulation of these infections during choir rehearsals, the survey of particle emissions and the assessment of aerosols in rooms are key elements in the risk management of ensemble and choir singing in enclosed rooms^15–17^.

The measuring method used (laser particle counter) provides a high accuracy concerning the absolute number of particles above 0.3 µm in size and the size of these particles because sources of interference have been reduced to a minimum. Furthermore, the suitability of the peripheral test setup could be proven within the scope of baseline measurements. If a participant sat in front of the entrance to the glass tube, wearing cleanroom clothing, minimizing movement, and holding their breath, only a few particles were detected. This proves that the detected particles for the different test conditions exclusively originate from the respiratory tract. Further, by choosing a constant airflow of 400 m^3^/h in the glass tube, which is much larger than the maximum airflow through the participant’s open lips, the quality of our results is rather independent of the expiratory airflow linked to the chosen test condition. A limitation of our experimental setup is a very high dilution ratio for low particle emission rates, resulting possibly in an underestimation by zero-counting. That means that realistic values for breathing might be higher than those determined in this study. Nevertheless, those values are lower than for speaking.

In addition to laser particle counters, several other methods have been established to determine aerosol emissions, for example, aerodynamic particle sizers (APS), and digital inline holography (DIH). These methods allow a more precise determination of the size of these particles. The disadvantage of APS measuring devices, however, is that due to the relatively low volume flow in the measuring device, the number of particles emitted by humans might be somewhat underestimated^13^ and the absolute number cannot be given for all conditions^14^. But assuming a homogeneous particle distribution at the location of the lips (and thereby the APS funnel), the APS is an appropriate instrument for assessing the particle concentration. The DIH method has the further advantage that the aerosol particles can be detected directly at the mouth^8^. Thus, their size can be well determined before evaporation. On the other hand, the limited spatial coverage might be a disadvantage, meaning that not all particles may be captured.

An alternative or supplemental method to investigate the size distribution of droplets during breathing, speaking, and singing is the imaging technique of Particle Image Velocimetry (PIV). This technique is based on high-resolution photos of the particles, which are illuminated, for example, with a laser light. Studies using PIV also show that more particles are emitted when speaking loudly than speaking with low voice^20^. However, mainly qualitative statements can be made here, due to several influencing factors. Size and number of particles can only be estimated, because of the background concentration of particles in the room and some drops can only be picked up in a blurred way. Cheng et al.^21^ measured particles of the sizes 1, 10, and 100 µm with PIV and high accuracy was shown for particles greater than 6 µm. This may be a reason why investigations of the size distribution of droplets with PIV led to significantly higher mean particle diameters^22^. Recent studies show that with PIV, particles on the order of 1 µm can be examined^19^. For particles, in the order of 0.3–25 µm, the laser particle counter used in cleanroom conditions offers higher accuracy in determining the number and size of particles.

It should be noted, that the initial velocity at the mouth, the constant airflow in the glass tube, and the distance of 0.81 m between the mouth and the laser particle counter lead to evaporated particle sizes as measured by the laser particle counter. According to Nicas et al.^23^, particles with an initial diameter of 20 µm shrink by a factor of 2, and according to We and Li^24^ particles up to 1000 µm shrink by a factor of 3 to the equilibrium diameter, nearly independently of the relative humidity in the room. The size of this final state is dependent on the amount of non-soluble residues, the humidity, and the residual respiratory fluid. This final state, is what is referred to as a particle in the context of this article.

Humidity has a high impact on the evaporation time of droplets. In general, the evaporation time is proportional to the square of the initial diameter^6,24^. Considering the greatest measured particle size of 25 µm in this study and a shrinking factor of 3, one can expect wet droplets of a maximum diameter of 75 µm.

Whereas a particle 15 µm in diameter evaporates in dry air in about 0.15 s to its resting state, the evaporation time increases at a relative humidity of 90% to 3.75 s (values were interpolated considering Table 1 in Wei and Li^24^). More than 80% of the particles measured in our study are equal to or smaller than 1 µm (3 µm unevaporated). For this particle size, evaporation times are on the order of 0.006 s in dry air, and 0.08 s in moist air. Additionally, because of the small initial size of about 15 µm in the maximum of the particles, no splitting into smaller particles happens which would influence the measured number of particles.

Based on the observed size distributions, sampling relative humidity, typical evaporation timescales associated with respiratory particles and droplets, and the flowrate through the glass sampling tube, the size of the particles measured represents aerosols which distribute in an environment and not as they are emitted by a participant directly at the mouth. Thus, the droplet nuclei represent a realistic measure for possible carrier particles for viruses, but are not associated with the number of viruses.

Since the aerosols emitted during breathing, speaking^20,25,26^, and singing^13,14^ are mainly $<1$ µm in size, it cannot be assumed that they sink quickly to the ground. It had been shown, that the retention time was in the range of minutes to hours and the sink rate is on the order of $<1$ mm/s^6,24,27^. The determined order of magnitude of the particle size of this study is significantly lower than the results of Loudon and Roberts^28^, where the particle emission during singing was also investigated. In this study, the estimated particle size during singing was determined with  68 µm in median. Furthermore, in the same study, the sizes of the emitted particles for speaking were determined to be 81 µm. Differences to our data might be reasoned in the difference in instrumentation. In their approach, larger droplets were easily countable by microscope making those more important. On the other hand, in our experimental setup, the detection of particles greater than 25 µm was not possible. For that reason, we cannot measure the emission of particles of that size for the investigated conditions within our study.

With regard to the size of emitted particles, one was able to show that they are distinctively smaller than 10 µm during speaking and breathing^20,29^. Recent studies using an aerodynamic particle sizer^13,14,20,25^ show similar size distributions of the emitted particles for the different conditions which was confirmed by our study. Regarding the absolute values of emission rates and particle concentrations for breathing, our data were in the same order of magnitude as reported recently^13,14,20,25^. For all other conditions (speaking, singing, and singing a sustained vowel), the data from this study show that increased intensity of phonation results in higher deviations regarding former results. Phonation is defined as sounds caused by the oscillating vocal folds in humans’ larynx driven by the airflow expelled from the lungs^30^. More precisely, the results show greater values than reported for speaking and singing (see Table 4). Reasons for this discrepancy might be mainly sourced by the limits of the internal airflow of the APS as argued by Alsved et al.^13^. Considering the particle-free environment in the cleanroom including zero-emission caused by the clothing, in reality, the discrepancy in the results could be even slightly larger. Similar to Gregson et al.^14^, we also observed a wide person-to-person variability. Particle concentrations reported in the literature are related to the (constant) airflow within the measurement device and not to the airflow at the lips. It should be noted, that for this study, transferring the emission rates into particle concentrations, an airflow at the lips of 5.9 l/min for breathing, and 12.6 l/min for all other conditions was used as determined for classical singers (based on the supplemental data given by Salomoni et al.^31^). Whereas aerosol particles, greater in diameter than the size of a virus (about 0.08 µm in case of SARS-CoV-2^32^), are a potential carrier for these infectious viruses, not only emission enhancement between the tasks is of interest but also their absolute number. This number of aerosols likely to stay airborne because of their size is highly relevant for airborne transmission studies and estimating the infection risk. In contrast to particle concentrations, emission rates as determined within this study can directly be applied in infection risk models for airborne viral transmission. It means that no assumption of not simultaneously measured airflow is necessary at all.

**Table 4:** Comparison of emission rates (in particles/sec) and particle concentrations (in particles/cm^3^) determined in this study with previously reported data.

Condition	This study (particles/sec)	This study (particles/cm^3^)	Alsved et al.^13^ (particles/sec)	Gregson et al.^14^ (particles/cm^3^)	Asadi et al.^20^, median from their Fig. 5 (particles/cm^3^)	Morawska et al.^25^, their Fig. 5 (particles/cm^3^)
**Breathing**	5	0.05	135	0.28	$\le$0.02	0.098
**Speaking**						
Normal loudn.	66	0.31	270	0.1	$\approx$0.07	0.32–1.088
Medium loudn.	–	–	–	0.22	$\approx$0.18	–
Loud	–	–	570	1.3	$\approx$0.32	–
**Singing**						
Normal loudn.	–	–	690	0.19	–	–
Medium loudn.	1537	7.32	980	0.52	–	–
Loud	–	–	–	2.0	–	–
Exaggerated loudn.	–	–	1480	–	–	–
**Sustained phonation**						
Piano	59	0.28	–	–	–	–
Mezzo-forte	589	2.80	–	–	–	–
Forte	2472	11.77	–	0.91	–	–

The particle concentrations for our study were derived by dividing our emission rates with an airflow of 5.9 l/min for breathing and with an airflow of 12.6 l/min for all other conditions (see Salomoni et al.^31^).

The present study confirms that higher emission rates of aerosols are produced during singing in comparison to speaking and breathing. A higher emission rate for speaking compared to breathing and an increase of emission rates with raising vocal loudness was found^20^. It should be noted, that $L_{AF_{MAX}}$, determined for this study, is effectively connected to the prominent peak value of the sound pressure level of test condition speaking and singing in experiment I. For the sustained vowels (experiment II), $L_{AF_{MAX}}$ represents a good approximation of its time-independent equivalent. Nevertheless, this quantity clearly shows the different sound pressure levels of each test condition (see Figs. 2 and 3).

However, phonation of sustained vowels, characterized by a periodic collision of the vocal folds correlating with pitch, does not reflect the ordinary situation in choral singing. Here, the order of consonants and vowels alternate in a sung passage and are interrupted by pauses. Therefore, in the present study, a sequence of 50 seconds of the choir piece “Abschied vom Walde” by Felix Mendelssohn Bartholdy was selected. Each line of the four-part choral movement was sung by the individually appropriate voice classification (soprano, alto, tenor, baritone). These data were compared with the tasks ’breathing’ and ’speaking’ (reading the standardized text corpus). Again, there is an increase in the emission rate for singing in comparison to speaking. Probably, this is due to the higher ratio of voiced segments to unvoiced segments and pauses, and increased sound pressure levels in singing. Our findings agree with the observation that voiced vocalizations lead to higher aerosol emissions, and the strong impact of vocal loudness^12,33^. We observe a median enhancement in emission rates for sustained phonation of 1.17 particles/sec, which is associated with a loudness difference of 1 dB (see Fig. 4). For speaking and singing, we determined an enhancement in emission rate of about 1.15 particles/sec for 1 dB loudness difference (not shown). These enhancements are equivalent to exponents of roughly 1.4 and 1.2 considering a power law regression for the emission rates and the sound pressure (in Pa). Comparing our determined exponents with previously reported values of 1.004 for a spoken /a/ and of 0.96 for a spoken text passage^20^ it seems likely, that the difference in particle emission between speaking and singing is about 72–86% driven by the difference in volume. The findings indicate that there is an additional but less influence of speaking versus singing. It should be noted, that the accuracy of these approximations is limited by different measurement methods (APS vs. LPC & SPL-Meter vs. Microphone). However, our data suppport the findings, that larger changes in aerosol emissions seem more related with volume than with the tasks^14^.

Apart from the influence of vocal loudness on the emission rate, we found increasing emission rate values associated with high pitch voice classifications. One reason for a stronger aerosol generation might be the higher frequency of the vibrating vocal folds. This counts both, for the soprano and alto line of the four-part choral movement and for the selected higher pitch for females during sustained phonation. These differences did not reach statistically significance, probably caused by the small sample size. With regard to the association of voice classification and gender, our findings confirm recent results, which did not find significant effects related to gender^20^.

The data presented here show no clear homogeneity within the cohort. For example, the emission rate determined for singing fluctuates by almost one order of magnitude. Also, the increase of $P_M$ between singing and speaking fluctuates by almost two orders of magnitude. Thus, the aspect of high-emitters or super-emitters might be considered^20^.

It should be emphasized that the determined emission rates do not provide information about possible concentrations of SARS-CoV-2 viruses yet. However, there are reasons to assume that an increasing number of viral RNA will be emitted when the aerosol emission rate is increased. It should be noted, that the probability of containing an infectious virus is about 0.01% for a particle of a dry diameter of about 1 µm^6^). This probability increases with increasing particle size^34^.

Our determined emission rates can serve to estimate the probability of infection^34,35^. Further, these values can serve as the basis for a detailed numerical analysis of airborne transmission under different settings^8,36^ and the estimation of quanta^37,38^, where one quantum is defined as the minimum dose of infectious viruses causing an infection in 63.2% of susceptile persons^39^. It should be noted that in the course of the actual pandemic so far, numerous situations seem to be related to a high probability of aerogenic virus transmission, including choir rehearsals^15–17,40^. There is an overwhelming evidence of viable SARS-CoV-2 viruses in indoor air^2–5,41^. However, comprehensive information on the transmission quantity and survivability of SARS-CoV-2 viruses in aerosols is still missing^42^.

Therefore, the present study contributes to one component in the risk assessment of singing, which in turn is largely determined by the current prevalence. Finally, there is a lack of data on whether specific breathing characteristics of singing (deep inhalation, higher intrapulmonary pressures) influence the risk of transmission when singing loudly. In any case, the data should support all efforts to improve the risk management, especially in choir singing.

## Methods

### Participants

Eight singers (aged 22–62 years; professional choir experience between 1 and 34 years) of a professional chamber choir (RIAS Kammerchor Berlin) took part in the investigations. Two singers belonged to each of the different voice classifications: baritone (S1 & S2), tenor (S3 & S4), alto (S5 & S6), and soprano (S7 & S8). This study was conducted according to the ethical principles based on the WMA Declaration of Helsinki and to the current legal provisions. It was approved by the ethics committee of the Technische Universität Berlin (TU Berlin), and informed written consent was obtained from all participants. It should be noted, that the results for breathing and speaking tasks of the participants considered in this study, have already been analyzed within a larger cohort^26^. In order to allow a direct comparison with the data for singing, the data of this subgroup were reused and analyzed.

### Particle measurements

The investigations were carried out in a cleanroom at the Hermann Rietschel Institute of the TU Berlin.

The supply air was introduced via a vertical low-turbulence displacement flow over the entire ceiling area of $4.8 \times 4.8$ m$^2$. The supply air velocity was 0.3 m/s and thus prevented thermal lift. The exhaust air was also discharged from the room over the entire surface via a raised floor. The room temperature was $295.15 \pm 0.5$ K, the relative humidity was $40 \pm 2$% and the room had 15 Pa overpressure to the surrounding rooms.

**Figure 5:**
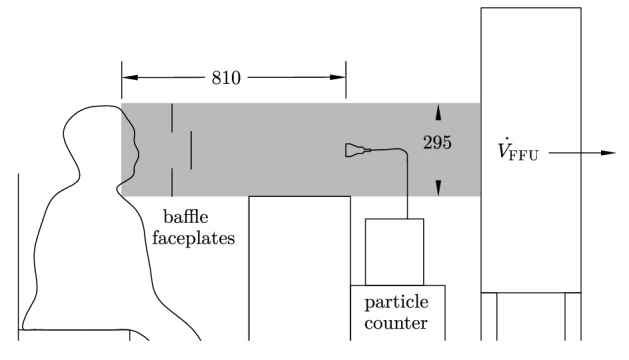
Schematic test setup with one person in cleanroom clothing whose exhaled air was recorded by the particle counter. The glass measuring section (gray colored area) was located on the suction side of a horizontally positioned Filter Fan Unit (FFU). All geometric dimensions are in mm (Figure adapted from Fig. 2 in Hartmann et al.^26^). (Figure created with cairo 1.15.10, http://cairographics.org).

The actual test stand was located in this clean environment (Fig. 5). It consisted of a glass tube, in which a constant airflow of 400 m^3^/h was generated by a filter fan unit (Ziehl-Abegg, Künzelsau, Deutschland). The measuring probe of a laser particle counter (Lighthouse Solair 3100 E, Lighthouse Worldwide Solutions, Fremont, CA) was placed centrally in the tube. The distance between mouth and laser particle counter was 0.81 m. To reach a homogenous particle distribution at the measurement position of the LPC, two baffle faceplates were incorporated^43^.

The particle counter was counting with a volume flow ${\dot{V}}_{PC}$ of 28.3 l/min, with a measuring time of 10 s each and detected particles in six size classes: $>0.3$–0.5 µm, $>0.5$–1.0 µm, $>1.0$–3.0 µm, $>3.0$–5.0 µm, $>5.0$–10 µm and $>10$–25 µm.

The emission rate $P_M$ presented in Figs. 2, 3 and 4 was computed based on the measured particle concentration $c_M$ and the volume flow through the filter fan unit (FFU) ${\dot{V}}_{FFU}$, i.e.

1 $$\begin{aligned} P_M=c_M \cdot {\dot{V}}_{FFU}. \end{aligned}$$

Volume flows of the particle counter and the FFU, as well as the measuring time, lead to an accuracy for $P_M$-values of 24 particles/sec for a measurement time of 10 s. This value decreases to 5 particles/sec with increased measuring time of 50 s for experiment I (see below).

To estimate sources of interference, such as background noise of particles in the room, as well as abrasion on the clothing and hair of the persons investigated, a baseline measurement was carried out at the beginning of the investigation. For particle reduction due to movement artifacts, the participants wore cleanroom clothing and a headgear with the sealing of the edges with adhesive tape, so that only eyes, nose, and mouth were uncovered.

In a baseline measurement, a count rate of the particle counter of $<1$ particles/5 min was determined within a measurement period of 10 min.

The counting efficiency for particles of the size 0.3 µm is $50 \pm 20$% and for particles of the size 0.5 µm it is $100 \pm 10$% according to ISO 21501-4. The measurements do not measure all the exhaled particles, but the particles in sizes above 0.3 µm (or 0.5 µm). To investigate how many particles were separated over the measuring distance, comparative measurements were made over a short distance from the particle counter. For this case, the particles were directly collected through a 150 mm high funnel while breathing and speaking and directed to the particle counter. The same size distribution was found as in the final configuration. For more detail on measurement setup see Mürbe et al.^43^.

### Audio measurements

The sound pressure level was determined using a calibrated sound level meter (CENTER 322_ Datalogger Sound Level Meter, Center Technologies, Houston, TX). During all measurements, the sound level meter was located approximately 60 cm anterior-laterally away from the mouth of the participants due to limited accessibility.

Furthermore, the high sensitivity of the particle counter did not allow a frontal positioning of the sound level meter inside the glass tube. Consequently, the determined levels cannot be considered as absolute levels but are lowered by a constant value of approx. 10 dB SPL. To account for the frequency-dependent sensitivity of human ears, the time- (Fast) and frequency-weighted (A-weighted) maximum sound pressure level $L_{AF_{MAX}}$ was determined. For sustained phonation, this quantity is approximately time-independent. Because of the time variability of the sound pressure levels for test conditions speaking and singing, $L_{AF_{MAX}}$ is correlated to local peaks of the sound power, and therefore of limited expressive power.

### Test conditions

The participants were sitting in front of the entry of the particle measurement setup. Two experiments were carried out:

- Experiment I: Comparison of three different test conditions
  - Breathing through the mouth
  - Reading a standardized text
  - Singing a line of a four-part choral movement
- Experiment II: Singing a sustained vowel (/aː/) at three loudness conditions
  - piano
  - mezzo-forte
  - forte

For experiment I, a time window of 50 s was analyzed for each of the experimental conditions. Further, for experiment II the analysis time window was set to 10 s. For reading in a comfortable loudness condition (Ib), the text “Der Nordwind und die Sonne” by Äsop was selected. To pass (Ic) the choral part of the song “Abschied vom Walde” by Felix Mendelssohn–Bartholdy was chosen. The participants were instructed to sing the line in their individual voice classification. Each of the tasks were repeated four times. If the participant faltered during the task or had to cough, the trial was terminated and repeated.

The following pitches were selected for experiment II: baritone: F3 (175 Hz), tenor: C4 (262 Hz), alto: F4 (349 Hz), and soprano: C5 (523 Hz). The intended level of vocal loudness was communicated to the participants using the terminology of musical dynamics with piano, mezzo-forte, and forte. These terms are familiar to professional singers.

The total measuring time for all tasks was about 30 min for each participant.

### Statistical analysis

Besides the description of the data, a confirmative analysis using linear mixed effect modeling was carried out^44–46^. Careful visual inspection of residual-plots and Q–Q-plots did reveal deviations from homoscedasticity and normality when using linear scaled emission rate values. Therefore, we calculated the log-transform of $P_M$ to overcome these problems (see also Gregson et al.^14^). It should be noted, that choosing log-transformed emission rates has no physical meaning. To suppress infinite values in the analyses caused by log-transform of zero entries, all values for $P_M$ were added by the increment of 5 particles/sec for experiment I, and 24 particles/sec for experiment II. As mentioned above, these values correspond to the smallest non-zero values that our paradigm delivers.

Finally, a linear mixed-effects analysis of the relationship between the dependent variable $\text {log}_{10}P_M$ and the independent variables test condition, voice classification, and participant was performed using the freely available software package R^47^ including the package lmerTest^48^. We tested, if test condition and voice classification effect the emission rates. Therefore, test condition and voice classification were incorporated as fixed effects into the model. Intercepts for participants were incorporated as random effects concerning the conditional dependence of the repetitions of each task (see Supplementary Data S1 for theoretical aspects). To test significance, the P-values were obtained by using Satterthwaite’s degree of freedom method. Linear mixed models were fit by restricted maximum likelihood.

## Supplementary Information

Supplementary Information.

## Data Availability

All data for this study and the R-code is available in the Supplementary Data S1.
