# Supplementary material for: Aerosol emission in professional singing of classical music
Source: Sci Rep. 2021 Jul 21;11:14861. doi: 10.1038/s41598-021-93281-x (PMC8295351; doi:10.1038/s41598-021-93281-x)

Data analysis

"Aerosol emission in professional singing of classical music"

Dirk Mürbe[1], Martin Kriegel[2], Julia Lange[2], Hansjörg Rotheudt[2], Mario Fleischer[1]

[1]Charité – Universitätsmedizin Berlin, Department of Audiology and Phoniatrics, Berlin, Germany
[2]Technische Universität Berlin, Hermann-Rietschel-Institut, Berlin, Germany

E-mail - dirk.muerbe@charite.de

Contents

1 Loading data **2**

2 Theory **2**

3 Experiment I: Comparing breathing, speaking and singing **3**
3.1 Select data . . . 3
3.2 Linear mixed effect modeling (ID as random effect, voice classification and condition as fixed effect) . . . 3
3.2.1 Non log-transformed emission rate as dependent variable . . . 3
3.2.2 Log-transformed emission rate as dependent variable . . . 4
3.3 Linear or log-transformed scaled emission rate – Deviation from normality and homoscedasticity? 4
3.4 Modell summary (log-transformed emission rates) . . . 5

4 Experiment II: Comparing piano, mezzoforte and forte **6**
4.1 Select data . . . 6
4.2 Linear mixed effect modeling (ID as random effect, voice classification and condition as fixed effect) . . . 7
4.2.1 Non log-transformed emission rate as dependent variable . . . 7
4.2.2 Log-transformed emission rate as dependent variable . . . 7
4.3 Linear or log-transformed scaled emission rate – Deviation from normality and homoscedasticity? 7
4.4 Modell summary (log-transformed emission rates) . . . 8

5 Plots of the data **10**

```
rm(list = ls())
library(lmerTest)
```

```
## Lade nötiges Paket: lme4

## Lade nötiges Paket: Matrix

##
## Attache Paket: 'lmerTest'

## Das folgende Objekt ist maskiert 'package:lme4':
##
##     lmer

## Das folgende Objekt ist maskiert 'package:stats':
```

```
##
##     step
```

```
library(dplyr)
```

```
##
## Attache Paket: 'dplyr'
## Die folgenden Objekte sind maskiert von 'package:stats':
##
##     filter, lag
## Die folgenden Objekte sind maskiert von 'package:base':
##
##     intersect, setdiff, setequal, union
```

```
library(ggResidpanel)
library(jtools)
library(ggplot2)
library(gridExtra)
```

```
##
## Attache Paket: 'gridExtra'
## Das folgende Objekt ist maskiert 'package:dplyr':
##
##     combine
```

```
library(latex2exp)
```

1 Loading data

Set working directory according to your file hierarchy. The file "data.csv" is part of the supplemental data.

```
orig.data <- read.csv('data.csv')
```

2 Theory

Linear mixed-effect modeling (LMEM) is a flexible approach for confirmatory analysis of multi-parametric data taking into account the lack of independence for repeated measurements. For this study, we considered the test condition (either breathing, speaking, and singing of experiment I, or piano, mezzo-forte, and forte for experiment II, respectively) and voice classification (baritone, tenor, alto, and soprano for both experiments) as fixed effects in our model. Further, we considered the participants' ID as a random effect. Both test condition and voice classification are independent variables, where the observations of emission rate (on log-scale and linear-scale) is the dependent variable. To ensure both stability as well as ease of interpretation, the chosen model structure does not taking into account the interaction of voice classification and test condition. Moreover, there is no physiological basis for this interaction. Altogether, the (general) mathematical notation for our chosen models can be given as:

$$y_{ij} = \underbrace{\beta_0 + \beta_1 \cdot x_{ij}^{\text{voice classification}} + \beta_2 \cdot x_{ij}^{\text{test condition}}}_{\text{voice classification test condition as fixed effects}} + \underbrace{S_{i0}}_{\substack{\text{participants' deviation as} \\ \text{normally distributed random effects} \\ \text{with zero mean}}} + \underbrace{e_{ij}}_{\text{residual errors}}$$

Here, y_{ij} is the response of participant i to the jth observation to the value of the explanatory variables voice classification ($x_{ij}^{\text{voice classification}}$), and test condition ($x_{ij}^{\text{test condition}}$). β_0 is the baseline level via fixed effects of these explanatory variables, and β_1 and β_2 the slopes of these variables. The normally distributed residual error is denoted with e_{ij}, and S_{i0} describes the normally distributed (with zero mean) deviation from β_0 for participant i (random intercepts).

The aim of the analysis is to find the optimal parameters for the fixed effects (β_0, β_1, and β_2), the variance-covariance matrix for the random effects (S_{i0}) and the variance of the residual errors e_{ij} to fit the data. Within this study, the analysis was carried out using the lmerTest-package. The associated lmer-Syntax to describe the equation above can be given as:

$$P_M \sim \text{VoiceClassification} + \text{Condition} + (1|\text{ID})$$

or, for the log-transformed data to

$$\log_{10} P_M \sim \text{VoiceClassification} + \text{Condition} + (1|\text{ID}).$$

Here, voice classification, and test conditions are the independent explanatory variables. ID is the unique identifier of each participant, and P_M are the measured particle emission rates. Whether using the log-transformed emission rates ($\log_{10} P_M$) or the linear-scaled ones (P_M) is discussed in the following sections. Further details can be found in Barr et al. (2013), http://dx.doi.org/10.1016/j.jml.2012.11.001 and Bates et al. (2015), https://doi.org/10.18637/jss.v067.i01.

3 Experiment I: Comparing breathing, speaking and singing

3.1 Select data

```
data.exp.I <- dplyr::filter(orig.data,
                             Condition == 'breathing'
                             | Condition == 'speaking'
                             | Condition == 'singing'
                            )
data.exp.I$Condition <- factor(data.exp.I$Condition,
                               levels = c('speaking','breathing','singing'))
data.exp.I$VoiceClassification <- factor(data.exp.I$VoiceClassification,
                               levels = c('Baritone','Tenor','Alto','Soprano'))
```

3.2 Linear mixed effect modeling (ID as random effect, voice classification and condition as fixed effect)

3.2.1 Non log-transformed emission rate as dependent variable

```
data.exp.I.model.ConditionVoiceClassification <-
                  lmer(PM.cum.mum~VoiceClassification+Condition
                   +(1|ID),
                  data.exp.I,
                  REML=TRUE)
```

3.2.2 Log-transformed emission rate as dependent variable

Shifting the database by the smallest non-zero value to avoid trouble caused by log(0).

```
data.exp.I.PM.tmp <- data.exp.I$PM.cum.mum
data.exp.I.PM.tmp.min <- min(data.exp.I.PM.tmp[data.exp.I.PM.tmp>0])
data.exp.I.PM.tmp.shift <- data.exp.I.PM.tmp+data.exp.I.PM.tmp.min
data.exp.I$PM.cum.mum.log = log10(data.exp.I.PM.tmp.shift)

data.exp.I.log.model.ConditionVoiceClassification <-
                  lmer(PM.cum.mum.log~VoiceClassification+Condition
                   +(1|ID),
                  data.exp.I,
                  REML=TRUE)
```

3.3 Linear or log-transformed scaled emission rate – Deviation from normality and homoscedasticity?

```
## `geom_smooth()` using formula 'y ~ x'
## `geom_smooth()` using formula 'y ~ x'
```

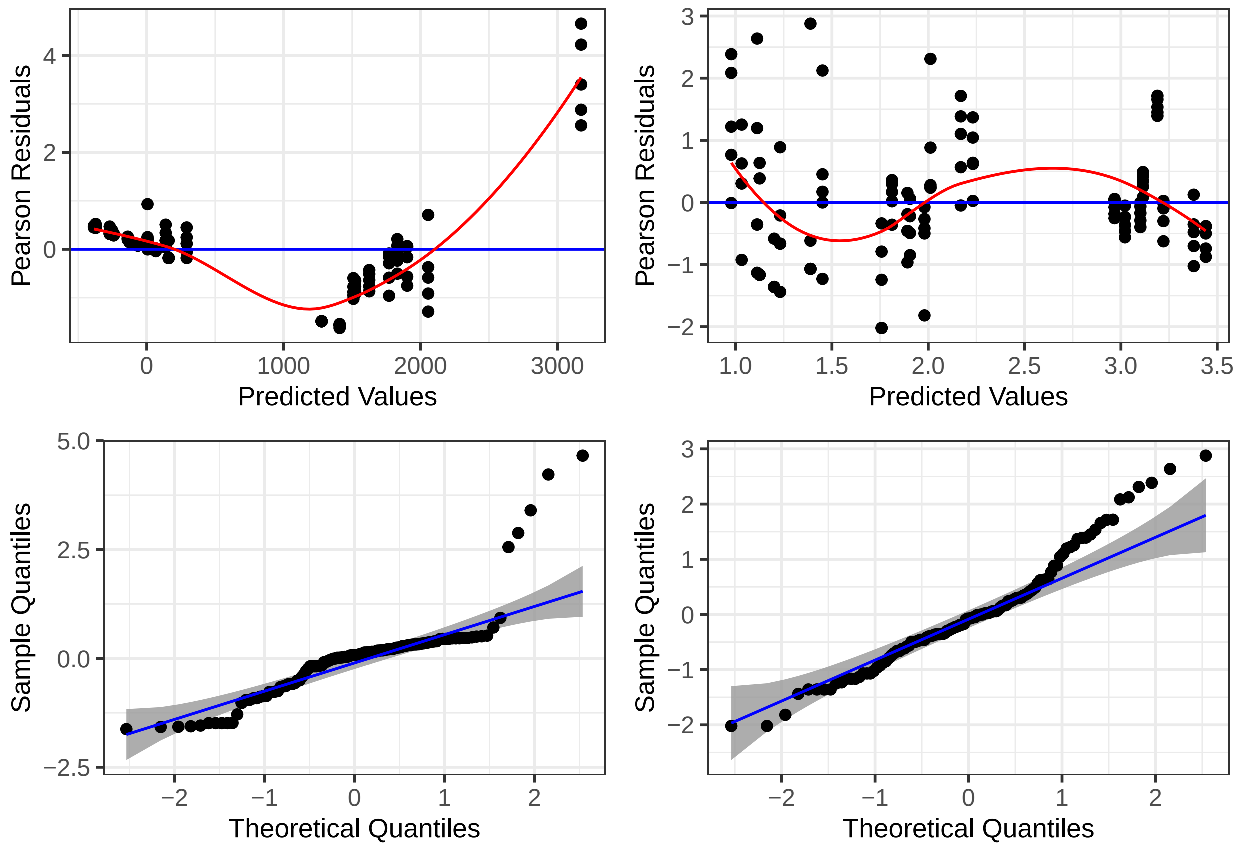

1) Both QQ-Plots and Residuals forces the usage of log-valued emission rate in the analysis
2) Normality and homoscedasticity is not (that) violated for log-valued emission rate

3.4 Modell summary (log-transformed emission rates)

```
summ(data.exp.I.log.model.ConditionVoiceClassification, digits = 3)
```

```
## Registered S3 methods overwritten by 'broom':
##   method            from
##   tidy.glht         jtools
##   tidy.summary.glht jtools
```

Observations	120
Dependent variable	PM.cum.mum.log
Type	Mixed effects linear regression

AIC	148.828
BIC	171.128
Pseudo-R^2 (fixed effects)	0.795
Pseudo-R^2 (total)	0.826

Fixed Effects					
	Est.	S.E.	t val.	d.f.	p
(Intercept)	1.785	0.145	12.276	5.147	0.000
VoiceClassificationTenor	0.167	0.193	0.865	4.000	0.436
VoiceClassificationAlto	0.290	0.193	1.501	4.000	0.208
VoiceClassificationSoprano	0.283	0.193	1.468	4.000	0.216
Conditionbreathing	-0.780	0.087	-8.992	110.000	0.000
Conditionsinging	1.209	0.087	13.934	110.000	0.000

p values calculated using Satterthwaite d.f.

Random Effects		
Group	Parameter	Std. Dev.
ID	(Intercept)	0.165
Residual		0.388

Grouping Variables		
Group	# groups	ICC
ID	8	0.153

```
plot_summs(data.exp.I.log.model.ConditionVoiceClassification,
           scale = TRUE, plot.distributions = TRUE, inner_ci_level = .95)
```

```
## Lade nötigen Namensraum: broom.mixed
```

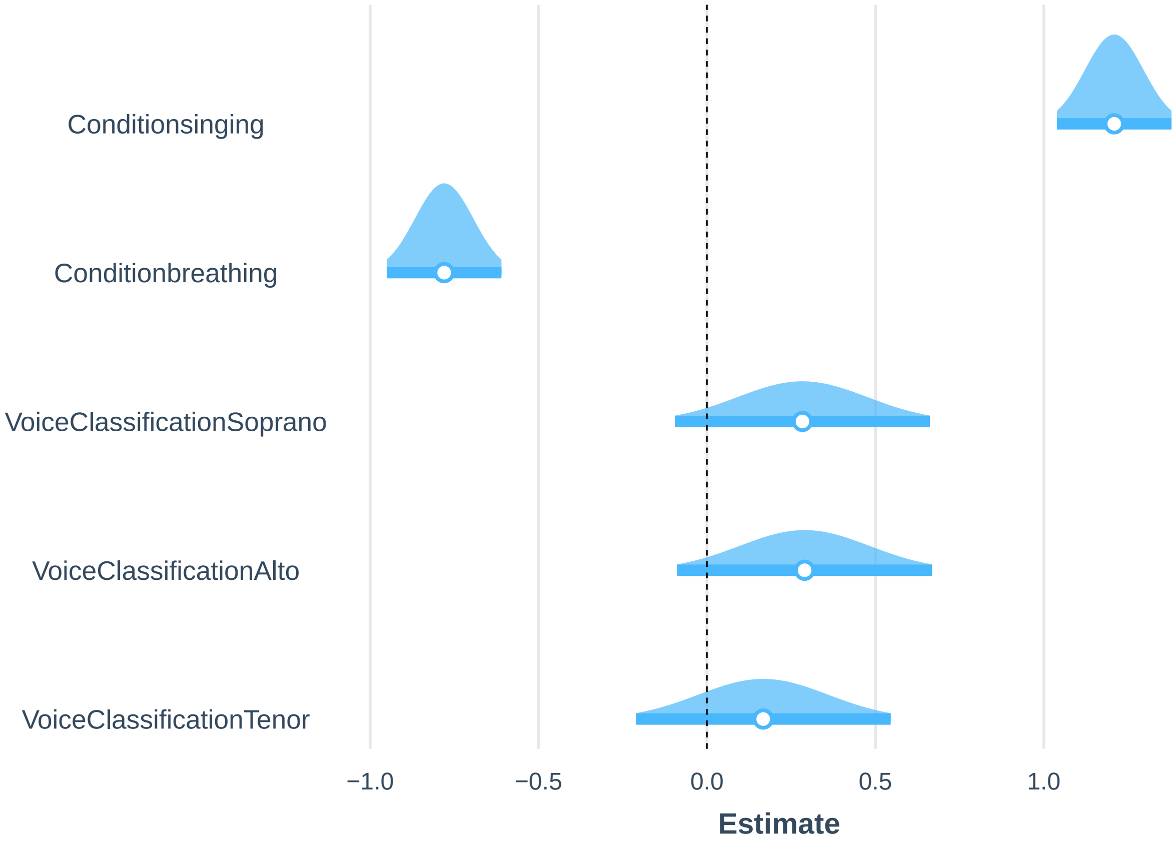

1) Condition affected $\log(P_M)$, increasing it by about 0.78 ± 0.09 (standard errors) from breathing to speaking (statistically significant, $p < .001$) and by about 1.21 ± 0.09 from speaking to singing (statistically significant, $p < .001$)
2) Voice classification affected $\log(P_M)$, increasing it by about 0.17 ± 0.19 (standard errors) from Baritone to Tenor (not statistically significant), by about 0.29 ± 0.19 (standard errors) from Baritone to Alto (not statistically significant), and by about 0.28 ± 0.19 (standard errors) from Baritone to Soprano (not statistically significant)
3) The intraclass correlation coefficient (ICC) for ID is about 15%

4 Experiment II: Comparing piano, mezzoforte and forte

4.1 Select data

```
data.exp.II <- dplyr::filter(orig.data,
                             Condition == 'piano'
                             | Condition == 'mezzoforte'
                             | Condition == 'forte'
                            )
data.exp.II$Condition <- factor(data.exp.II$Condition,
                                levels = c('mezzoforte','piano','forte'))
data.exp.II$VoiceClassification <- factor(data.exp.II$VoiceClassification,
                                levels = c('Baritone','Tenor','Alto','Soprano'))
```

4.2 Linear mixed effect modeling (ID as random effect, voice classification and condition as fixed effect)

4.2.1 Non log-transformed emission rate as dependent variable

```
data.exp.II.model.ConditionVoiceClassification <-
                  lmer(PM.cum.mum~VoiceClassification+Condition
                   +(1|ID),
                  data.exp.II,
                  REML=TRUE)
```

4.2.2 Log-transformed emission rate as dependent variable

Shifting the database by the smallest non-zero value to avoid trouble caused by log(0).

```
data.exp.II.PM.tmp <- data.exp.II$PM.cum.mum
data.exp.II.PM.tmp.min <- min(data.exp.II.PM.tmp[data.exp.II.PM.tmp>0])
data.exp.II.PM.tmp.shift <- data.exp.II.PM.tmp+data.exp.II.PM.tmp.min
data.exp.II$PM.cum.mum.log = log10(data.exp.II.PM.tmp.shift)

data.exp.II.log.model.ConditionVoiceClassification <-
                  lmer(PM.cum.mum.log~VoiceClassification+Condition
                   +(1|ID),
                  data.exp.II,
                  REML=TRUE)
```

4.3 Linear or log-transformed scaled emission rate – Deviation from normality and homoscedasticity?

```
## `geom_smooth()` using formula 'y ~ x'
## `geom_smooth()` using formula 'y ~ x'
```

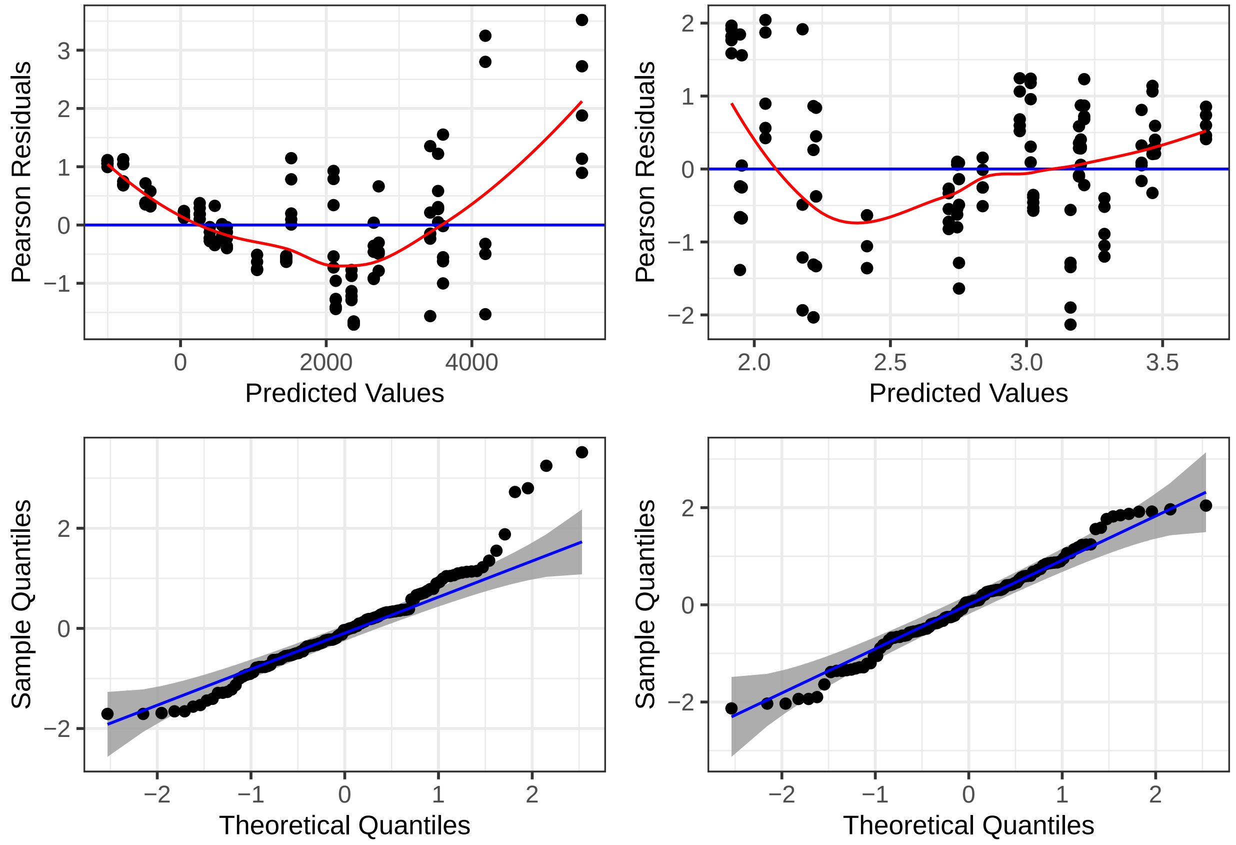

1) Both QQ-Plots and Residuals forces the usage of log-valued emission rate in the analysis
2) Normality and homoscedasticity is not (that) violated for log-valued emission rate

4.4 Modell summary (log-transformed emission rates)

```
summ(data.exp.II.log.model.ConditionVoiceClassification, digits = 3)
```

Observations	120
Dependent variable	PM.cum.mum.log
Type	Mixed effects linear regression

AIC	164.453
BIC	186.753
Pseudo-R^2 (fixed effects)	0.583
Pseudo-R^2 (total)	0.645

```
plot_summs(data.exp.II.log.model.ConditionVoiceClassification,
           scale = TRUE, plot.distributions = TRUE, inner_ci_level = .95)
```

Fixed Effects					
	Est.	S.E.	t val.	d.f.	p
(Intercept)	2.777	0.154	18.061	5.184	0.000
VoiceClassificationTenor	0.087	0.204	0.427	4.000	0.691
VoiceClassificationAlto	0.104	0.204	0.510	4.000	0.637
VoiceClassificationSoprano	0.342	0.204	1.678	4.000	0.169
Conditionpiano	-0.798	0.093	-8.582	110.000	0.000
Conditionforte	0.447	0.093	4.811	110.000	0.000

p values calculated using Satterthwaite d.f.

Random Effects		
Group	Parameter	Std. Dev.
ID	(Intercept)	0.173
Residual		0.416

Grouping Variables		
Group	# groups	ICC
ID	8	0.148

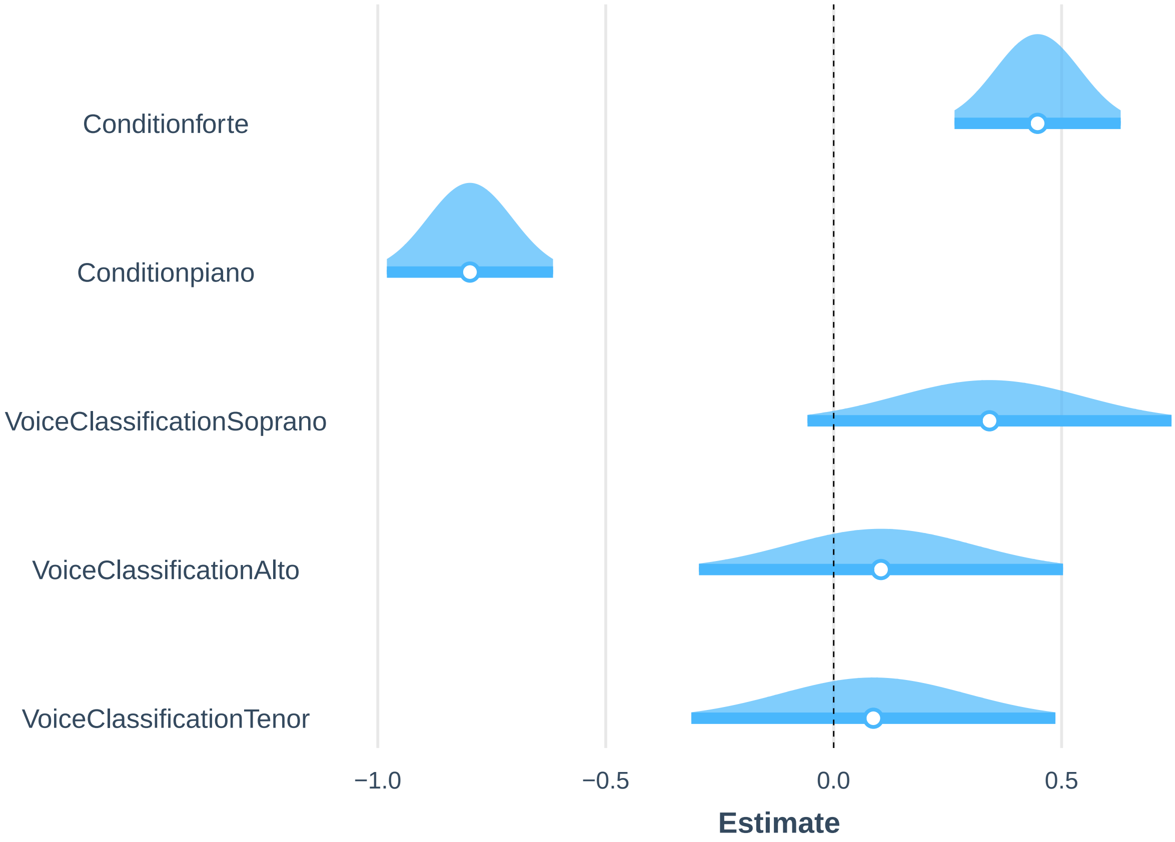

1) Condition affected $\log(P_M)$, increasing it by about 0.80 ± 0.09 (standard errors) from piano to mezzo-forte (statistically significant, $p < .001$) and by about 0.45 ± 0.09 from mezzo-forte to forte (statistically significant, $p < 2e - 16$)

2) Voice classification affected $\log(P_M)$, increasing it by about 0.09 ± 0.20 (standard errors) from Baritone to Tenor (not statistically significant), by about 0.10 ± 0.20 (standard errors) from Baritone to Alto (not statistically significant), and by about 0.34 ± 0.20 (standard errors) from Baritone to Soprano (not statistically significant)

3) The intraclass correlation coefficient (ICC) for ID is about 15%

5 Plots of the data

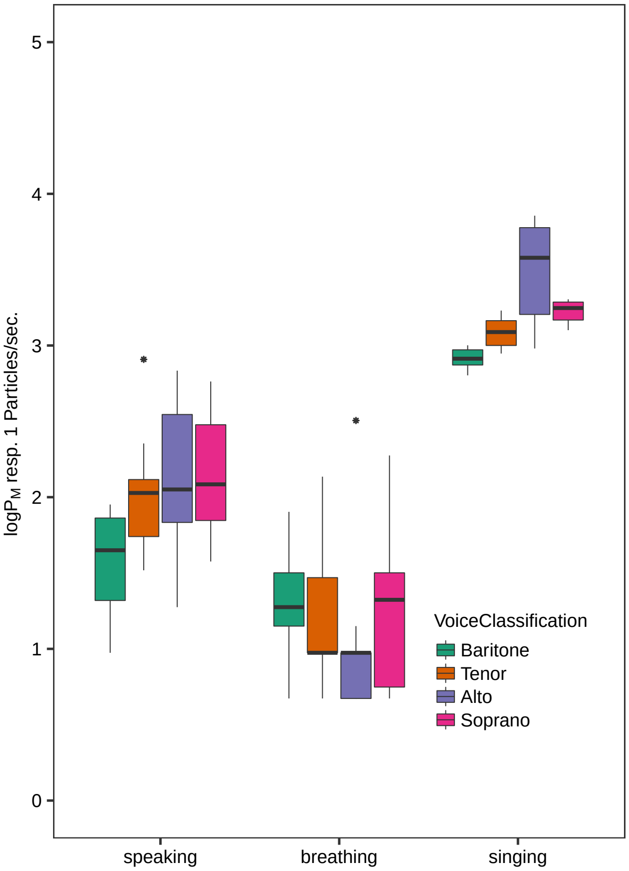

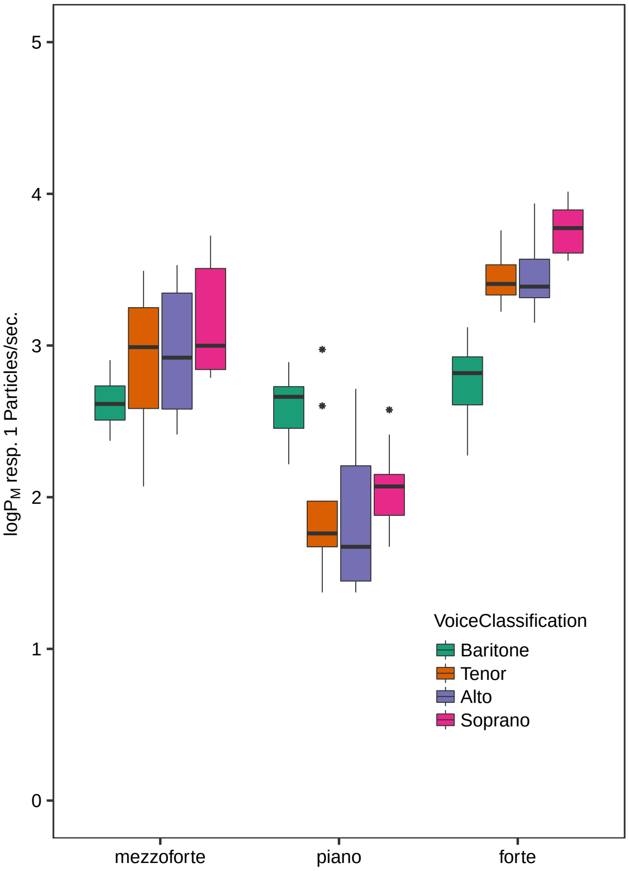

Supplement: Supplementary file 1 — Supplementary Information. [file 41598_2021_93281_MOESM1_ESM.zip › Muerbe_2021_singer_Sci_Rep_Statistic.pdf]
